# Advances in single-scan time-encoding magnetic resonance imaging

**DOI:** 10.1038/s41598-018-28460-4

**Published:** 2018-07-18

**Authors:** Sina Marhabaie, Geoffrey Bodenhausen, Philippe Pelupessy

**Affiliations:** Laboratoire des biomolécules, LBM, Département de chimie, École normale supérieure, PSL University, Sorbonne Université, CNRS, 75005 Paris, France

## Abstract

Time-encoding MRI is a single-scan method that uses traditional *k*-encoding only in one direction. In the orthogonal “time-encoding” direction, a string of echoes appears in an order that depends on the position of the corresponding spin packets. In one variant of time-encoding, this is achieved by using a series of selective pulses and appropriate gradients in both *k*-encoding and time-encoding directions. Although time-encoding offers some advantages over traditional single-scan Fourier methods such as echo planar imaging (EPI), the original time-encoding sequence also has some drawbacks that limit its applications. In this work, we show how one can improve several aspects of the original time-encoding sequence. By using an additional gradient pulse one can change the order in which the echoes appear, leading to identical echo times for all echoes, and hence to a uniform signal attenuation due to transverse relaxation and a reduction in average signal attenuation due to diffusion. By rearranging positive and negative gradients one can reduce the switching rate of the gradients. Furthermore, we show how one can implement time-encoding sequences in an interleaved fashion in order to reduce signal attenuation due to transverse relaxation and diffusion, while increasing the spatial resolution.

## Introduction

**Figure 1:**
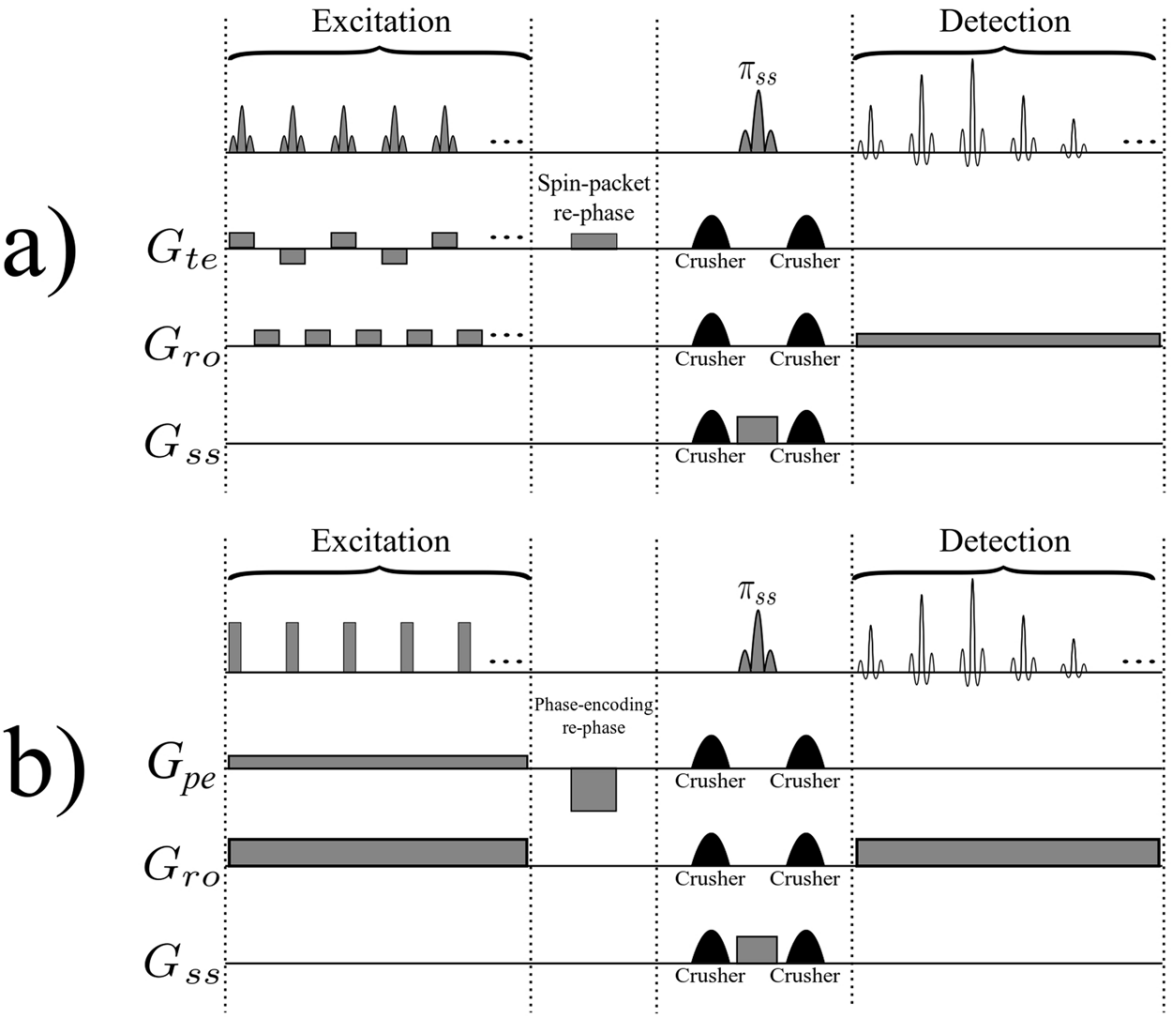
(**a**) The original hybrid time-encoding sequence proposed by Meyerand and Wong^2^, and (**b**) a typical BURST sequence. *G*_*ss*_, *G*_*pe*_, *G*_*ro*_, and *G*_*te*_ represent slice-selection, phase-encoding, readout, and time-encoding gradients. In both cases, the re-phasing gradients will have the effect of un-winding the phase accumulated during excitation. The detection blocks of these two sequences are similar. Note that in the original BURST sequence^15,16^ phase-encoding is done in the detection block of the sequence, whereas in the modified version of BURST^17^ shown in (**b**), phase-encoding is achieved in the excitation block of the sequence.

Time-encoding is a single-scan MRI technique that was introduced by Meyerand and co-workers in the 1990’s^1–5^. It is a hybrid MRI technique that uses traditional *k*-encoding only in one direction. In the orthogonal “time-encoding” direction, the timing of each echo depends on the position of the corresponding spin packet. In one variant of time-encoding^2^, this is achieved by using a series of selective pulses and appropriate gradients in both *k*-encoding and time-encoding directions. This is similar to line-scanning^6^ techniques that use traditional *k*-encoding in one direction only, while the second direction is retrieved through a series of parallel strips (or “lines” or “spin packets”) that are excited selectively. A one-dimensional image of each spin packet is obtained by using the traditional *k*-encoding method. These one-dimensional images are then arranged sequentially, resulting in a two-dimensional image. The original sequence used by Meyerand and Wong^2^ is shown in Figure 1. The sequence starts by the sequential selective excitation of *N*_*te*_ contiguous planes (spin packets), where *N*_*te*_ is the matrix size in the time-encoding direction, which is equal to the number of radiofrequency (*rf*) pulses in the excitation block. During excitation, the magnetization vectors in these planes are de-phased in the readout direction by the gradient pulses in the excitation block, while in the time-encoding direction the effects of positive and negative gradient pulses compensate each other. A gradient pulse, inserted between the excitation block and the π pulse, re-phases the phase twist imparted on the magnetization of these planes by the spin-packet-selective pulses. A slice perpendicular to the contiguous planes is then selected by a shaped π pulse. Since the readout gradient pulses applied in the excitation block have an additive effect, the spin packets will be re-phased one-by-one in the detection block, bringing about an echo for each spin packet, i.e., for each line. Therefore, to produce a two-dimensional image it is sufficient to arrange these echoes in a suitable array and perform a one-dimensional Fourier transformation on each echo.

Time-encoding^2–5^ can be considered as a SPatiotemporal ENcoding (SPEN^7,8^) technique^9–12^. Such MRI methods are based on the localization of a signal from a chosen volume of the sample, and the signal acquisition is done in such a way that there is a one-to-one correspondence between the signal intensity (usually recorded as a function of time) and the spin density. Consequently, in such methods the signal intensity itself looks like the object, and there is no need for any Fourier transformation to reconstruct the image (in the SPEN direction). Single-scan hybrid encoding techniques that use spatiotemporal encoding in one direction and *k*-encoding in the other have been shown to be superior to conventional single-scan methods that use *k*-encoding in both directions in suppressing the effects of frequency variations that may be caused by inhomogeneous magnetic fields, the presence of several chemical shifts, or any other frequency variation. They lead to images that are much less distorted than in conventional methods^13,14^.

Like other line-scanning techniques, time-encoding is less sensitive to motion, and can be particularly useful to study a dynamic system, e.g., for cardiac imaging^5^. In this method, each point in the raw data matrix corresponds to a single spin packet. As a result, the effects of motion are restricted to the intervals in which the motion has occurred^5^. Imagine a case in which the object moves during some time intervals while the object is being imaged, but remains steady in all other time intervals. In such a case, motional artefacts in time-encoding techniques are restricted to only those spin packets that have been recorded during the time intervals in which the object has moved, while the rest of the object remains free from artefacts. In contrast to time-encoding methods, each point of the raw data matrix obtained by *k*-encoding methods contains signals from the entire image. This means that the effects of motion spread out across the entire object even if the object moves only during a small part of the acquisition time, and remains stationary during most of the time.

An additional advantage of time-encoding methods is that aliasing does not occur in this method, even if the object being imaged is larger than the field of view (FOV). Using digital filters one can easily remove aliasing in the readout direction in both time-encoding and traditional *k*-encoding methods. However, removal of aliasing artefacts (“wrap-around artefacts”) in the phase-encoding direction in traditional *k*-encoding methods requires some special techniques, such as pre-saturation of areas that lie outside the field of view. In time-encoding techniques, on the other hand, wrap-around artefacts do not appear, since these techniques do not require any Fourier transformation in the time-encoding direction.

In the original time-encoding sequence, the echo times are different for different spin packets, leading to a non-uniform weighting of the signals. In this work we show how one can modify the original sequence to obtain identical echo times for all spin packets. Moreover, it will be shown that how one can reduce the gradient switching rate by rearranging the gradients. In addition, it is shown how one can implement the sequence in an interleaved fashion in order to reduce signal attenuation due to diffusion. Finally, we show that the sequences can be modified in such a way that all echoes are uniformly affected by inhomogeneous *T*_2_^*inh*^ effects, defined by 1/*T*_2_^*^ = 1/*T*_2_ + 1/*T*_2_^*inh*^. In the modified version, the sequence does not lead to non-uniform weighting of signals from different parts of the object. If *T*_2_^*inh*^ is position-independent, the attenuation of the signal of a voxel does not explicitly depend on the position of that voxel.

At this point, it worth drawing a comparison between time-encoding and BURST^15,16^ imaging techniques. Figure 1b shows a typical BURST sequence. Since only constant gradients are used, BURST methods have very low gradient switching rates. Because they use small flip angle pulses and a constant readout gradient, BURST methods bring about a series of equally spaced echoes that reflect equal intervals in *k*-space. These echoes are phase-encoded in the conventional manner, so that the image is obtained after two-dimensional Fourier transformation (2D-FT). The detection blocks for BURST and time-encoding sequences are similar: both techniques use a train of echoes for detection. In BURST these echoes are phase-encoded, whereas in hybrid time-encoding techniques they are time-encoded; that is each echo corresponds to a time at which the corresponding spin-packet has been excited.

Although BURST benefits from very low gradient switching rates, it suffers from low sensitivity, and apparently has not found widespread applications in clinical imaging so far^17^. Furthermore, since BURST is based on a Fourier transformation, it is more sensitive to field inhomogeneities and motion. Aliasing will occur in the phase-encoding direction if the object is larger than the FOV, which does not occur with time-encoding methods.

## Results and Discussion

### Uniform echo times

**Figure 2:**
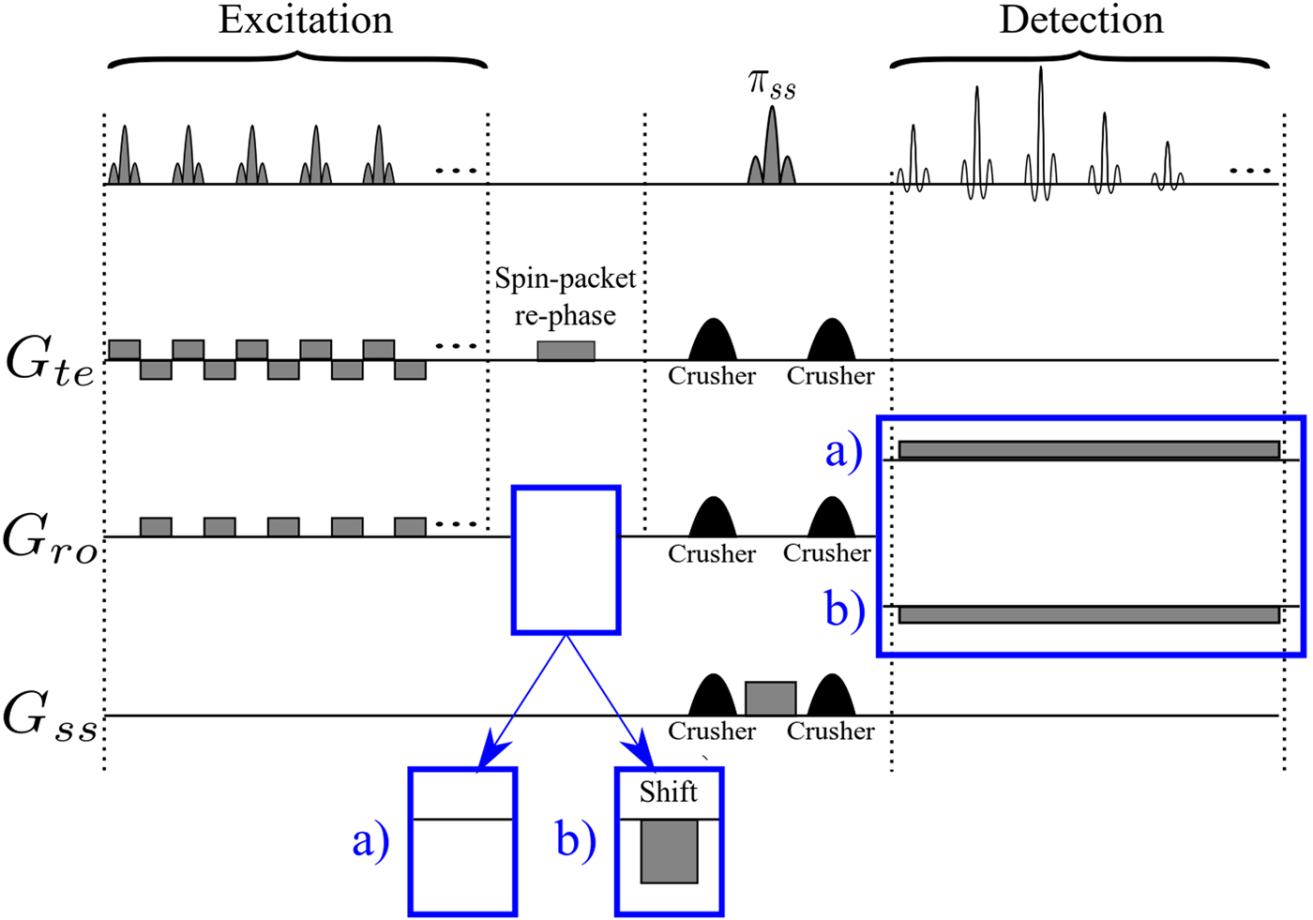
(**a**) Basic time-encoding sequence without re-ordering of the signals, and (**b**) modified time-encoding sequence with re-ordering of the signals. The spin-packet re-phasing gradient pulse unwinds the phases accumulated during the excitation of the spin packets in (**a**) and (**b**). The shift gradient in (**b**) reverses the order in which the signals of the spin packets appear, so that they occur in the same order as they were excited, leading to identical *TE* for all echoes in (**b**), in contrast to (**a**). The crusher gradients remove artefacts from the images. *G*_*ss*_, *G*_*ro*_, and *G*_*te*_ stand for slice-selection, readout, and time-encoding gradients, respectively.

**Figure 3:**
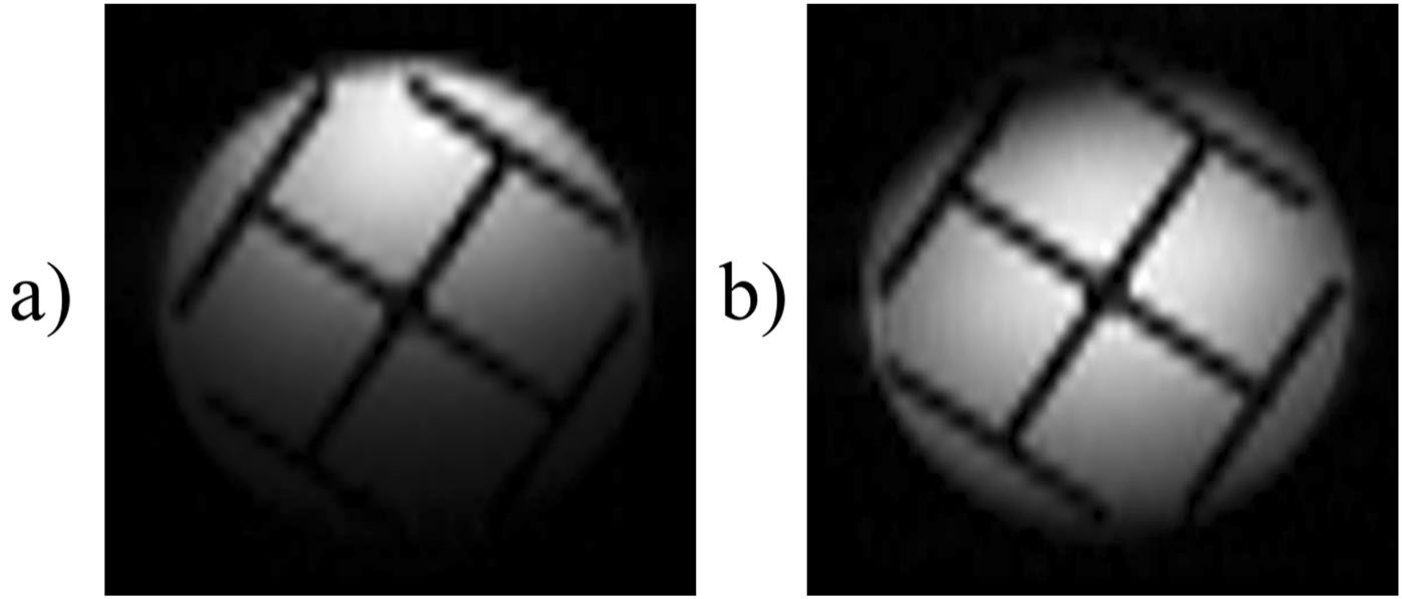
(**a**) Image of a phantom of a cross-shaped piece of plastic immersed in water obtained with the basic time-encoding sequence of Figure 2a, with different echo times for different echoes. (**b**) A time-encoding image obtained with the modified sequence of Figure 2b with identical echo times for all echoes. Both images have been recorded with a matrix size of 64 in the (horizontal) *k*-encoding direction, and 32 in the (vertical) time-encoding direction, an FOV of 27.0 × 27.0 mm, and a slice thickness of 1.0 mm. The average echo time was *TE* = 22.6 ms in (**a**), while *TE* = 22.6 ms for all echoes in (**b**). In both cases a readout bandwidth of 125 kHz, a total scan time 39.0 ms, and a Gaussian pulse with a duration of 256 µs and a flip angle of 90° were used for spin-packet selection. The calculated mean signal in (**b**) is 18% larger than in (**a**).

Figure 2a depicts a variant of the original time-encoding sequence proposed by Meyerand and Wong^2^. In this variant, the *rf* excitation pulses are only applied during positive time-encoding gradients, in contrast to Figure 1 where *rf* pulses are applied during both positive and negative gradients in the excitation sequence. Figure 2b represents a modified version where the echo times are the same for all echoes. The idea is to reverse the order of the echoes with respect to the original sequence, which can be done by inserting a shift gradient pulse. The area under this gradient pulse must be equal to the total area of all readout gradient pulses in the excitation block, plus half of the area of a single gradient pulse. Adding such a shift gradient pulse will exactly re-phase the first spin packet, whereas the re-phasing of the second spin packet will be slightly overdone, so that it will be slightly de-phased. The third spin packet will be even more de-phased, and so on. As a result, in contrast to the original sequence, spin packets that are excited earlier will also be detected earlier, and those which are excited later will be detected later. As a result of this modification, all echoes will have the same echo time *TE*, provided that the durations of the acquisition and excitation sequences are the same. Adding half the area of a single gradient pulse to the shift gradient pulse causes the echo of each spin packet to occur in the middle of the corresponding detection interval, thus increasing the signal-to-noise ratio. Figure 3a shows an image obtained by the sequence of Figure 2a, while Figure 3b was obtained by the improved sequence of Figure 2b. The phantom we used was a cross-shaped piece of plastic that was placed in a 25 mm O.D. glass tube filled with de-ionized water. Evidently, the modified image of Figure 3b has a better average signal-to-noise ratio (SNR) than Figure 3a. If a de-phasing interval τ is followed by an equal re-phasing interval, the signal loss due to diffusion is given by^18,19^:

1 $$s={s}_{0}\,\exp (-\,\frac{2}{3}D{{\rm{\gamma }}}^{2}{G}^{2}{{\rm{\tau }}}^{3})$$

where *s*_0_ and *s* are the signal amplitudes in the absence and presence of diffusion respectively, γ is the gyromagnetic ratio, *D* is the diffusion coefficient, τ is the duration of the de-phasing and re-phasing intervals, and *G* is the amplitude of the gradient. Therefore in Figure 3a, for those spin packets that are detected at the end of the sequence (the area at the bottom of the Figure 3a which corresponds to a long τ) the signal loss due to diffusion is large. In Figure 3b on the other hand, the signal loss due to diffusion behaves like in the RASER (Rapid Acquisition by Sequential Excitation and Refocusing) sequence^20,21^, where the areas at the bottom (and at the top) of the Figure 3b correspond to effective values for τ which are shorter than in Figure 3a. Consequently, in Figure 3b the average SNR is larger than in Figure 3a. Replacing the experimental values (*D* = 2.3 × 10^−9^ m^2^ s^−1^, γ*G ~* 4.0 × 10^6^ Hz m^−1^, τ ~ 0.044 s for Figure 3a, and, τ ~ 0.022 s for Figure 3b) in Equation 1 allows one to estimate the diffusional loss to be at worst 86% in Figure 3a (*s*/*s*_0_ ~ 0.14), and 23% in Figure 3b (*s*/*s*_0_ ~ 0.77)$.$

### The effects of excitation pulses with different shapes

**Figure 4:**
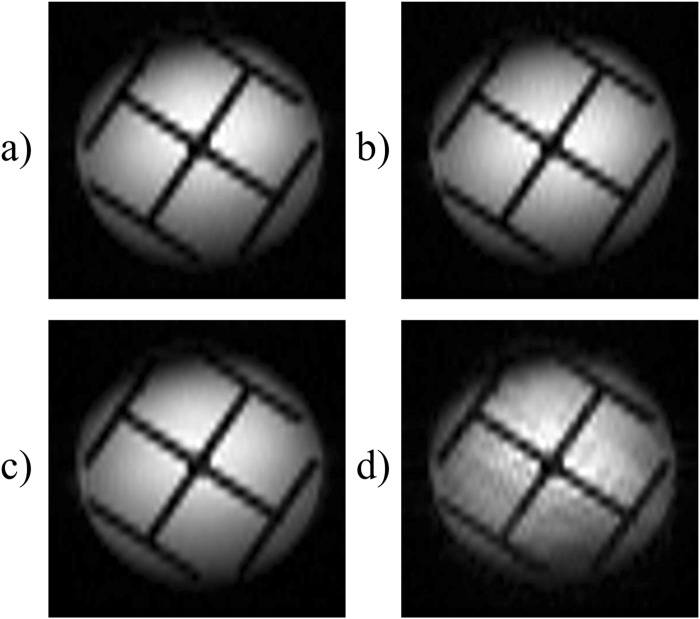
Four different images obtained using the sequence shown in Figure 2b with different excitation pulses. (**a**) Using a 45° “Sinc” excitation pulse truncated at the second zero-passages, and a time-encoding bandwidth *bw*_*te*_ = 496 kHz. (**b**) Using a 45° “Sinc” excitation pulse truncated at the third zero-passages, with *bw*_*te*_ = 744 kHz. (**c**) Using a 90° Gaussian excitation pulse, and *bw*_*te*_ = 310 kHz, (**d**) Using a 90° rectangular excitation pulse, and *bw*_*te*_ = 155 kHz. In all cases, the strength of the excitation gradient was adjusted to match the bandwidth of the different shaped pulses. All images were obtained with a total matrix size 64 in the *k*-encoding direction, and 32 in the time-encoding direction, an FOV of 27.0 × 27.0 mm, and *TE* = 22.6 ms. The lengths of the excitation pulses were 256 µs in all cases.

Figure 4 shows the effects of different shapes of the selective pulses used to excite the spin packets in the time-encoding dimension. All images were obtained by the sequence shown in Figure 2b. A “Sinc” excitation pulse extending over two cycles was used in Figure 4a, a “Sinc” excitation pulse with three cycles in Figure 4b, a Gaussian excitation pulse in Figure 4c, and a rectangular excitation pulse in Figure 4d. All four shaped pulses had the same length of 256 µs, but they gave rise to different excitation bandwidths. In each of the four experiments, the strength of the excitation gradient was adjusted to avoid overlaps between neighbouring spin packets. Although the pulse shapes obviously have some effects, the differences between the four images are not significant. However, the images of Figures 4c and d are to be preferred because they were obtained using a smaller bandwidth in the time-encoding direction and, consequently, weaker time-encoding gradient pulses.

### Interleaved spin packet selection

In all time-encoding sequences, the use of long and intense gradients will cause signal attenuation due to diffusion. To avoid this effect, one can shorten the entire sequence by reducing the number of pixels. This will of course deteriorate the resolution in the time-encoding direction. Alternatively, one can divide the experiment into several interleaved scans, where each scan uses a different set of frequencies to excite different interleaved spin packets. This reduces the length of each scan, thereby diminishing signal attenuation due to diffusion. In practice, to implement such interleaved sequences, the frequencies of the spin-packet-selective pulses can be chosen so that immediately adjacent spin packets are not excited in individual scans, but only excited in complementary scans in an interleaved fashion.

**Figure 5:**
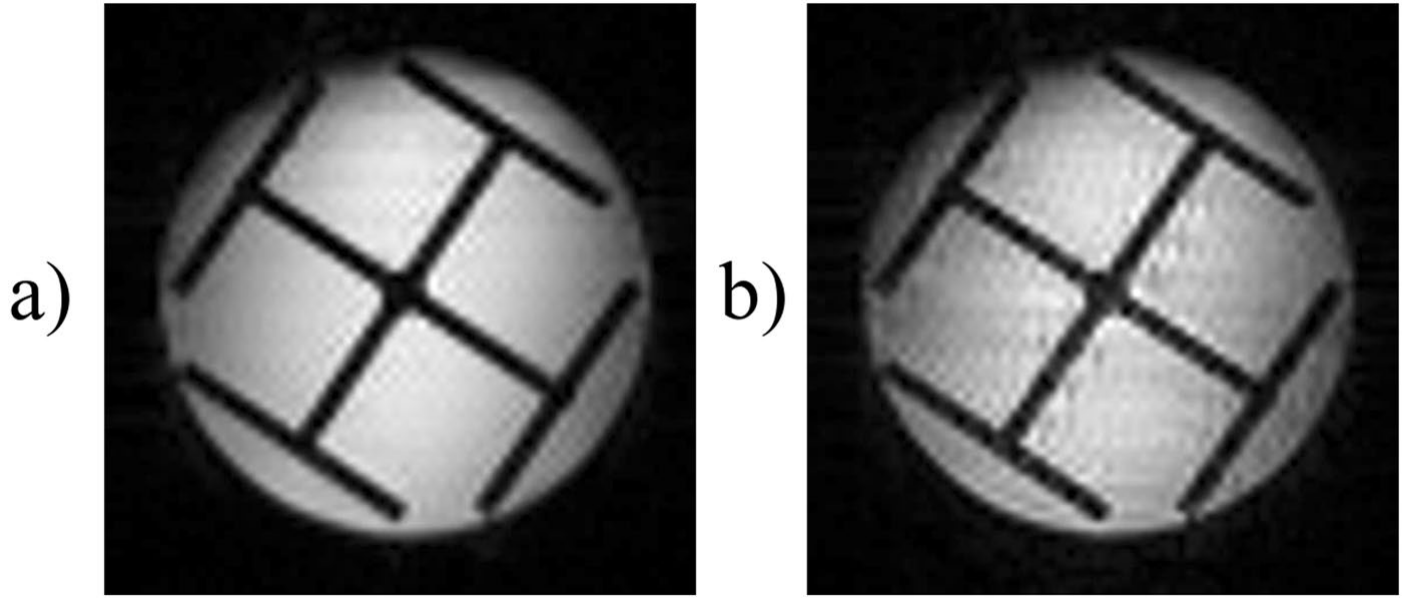
Two time-encoding images obtained using a four-fold interleaving of the excitation frequencies in the sequence of Figure 2b, with a matrix size of 64 × 64 (16 × 64 for individual scans), an FOV of 27.0 × 27.0 mm, and *TE* = 14.4 ms. Gaussian pulses were used for excitation in (**a**), and rectangular pulses in (**b**).

Figure 5 shows two images of the cross-shaped phantom obtained by the sequence of Figure 2b using a four-fold reduction of the length of the excitation and detection sequences combined with a four-fold interleaving. As can be seen, the signal attenuation near the top or bottom of Figures 3 and 4, which is due to diffusion, is greatly reduced in Figure 5.

### Reducing the gradient switching rate

**Figure 6:**
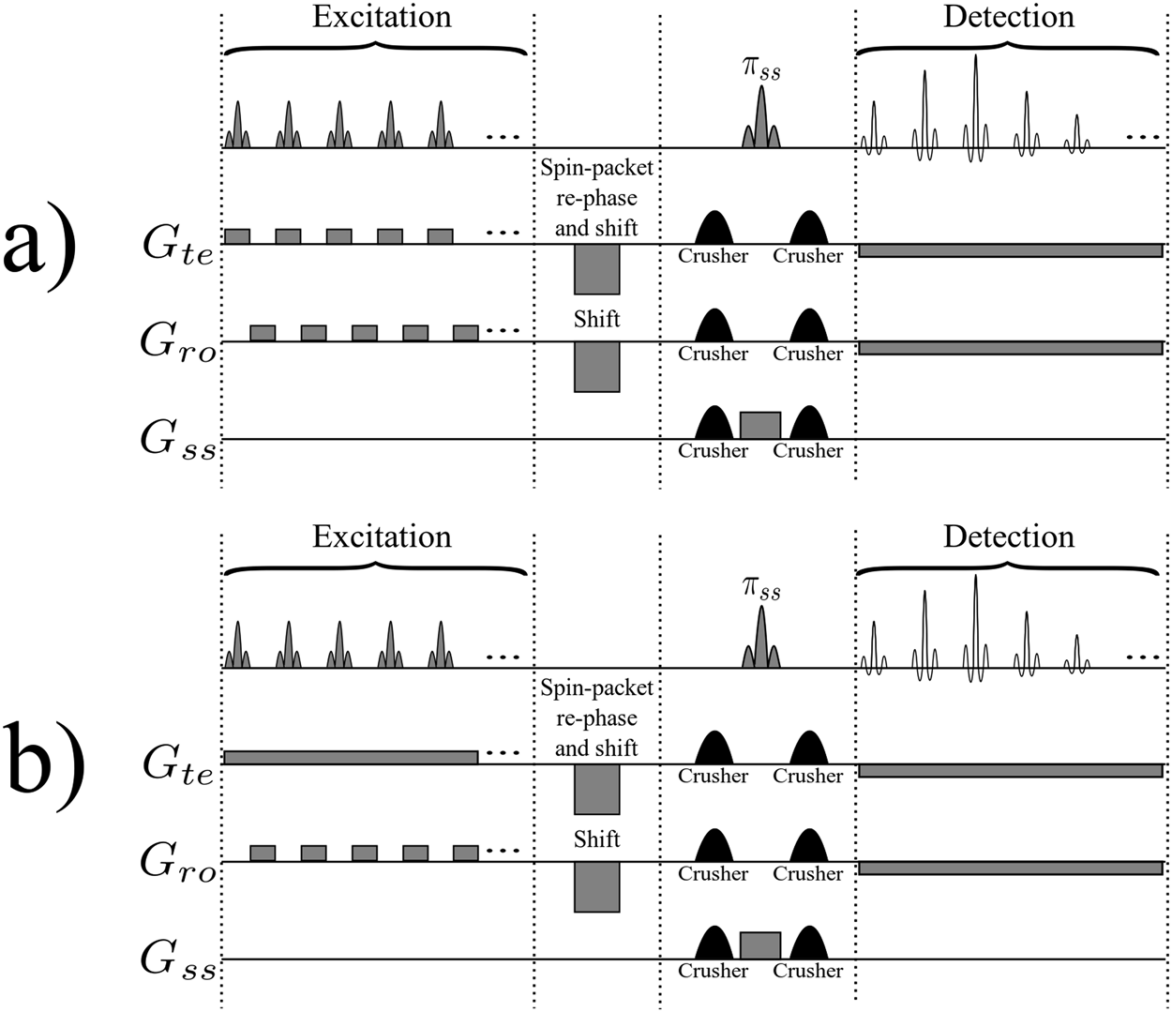
(**a**) Alternative hybrid time-encoding sequence in which the spin packets are re-phased in the detection sequence. (**b**) A decrease of the gradient switching rate can be achieved by replacing the spin-packet-selective gradient pulses in the *G*_*te*_ channel by a constant gradient. In both sequences, *G*_*ss*_, *G*_*ro*_, and *G*_*te*_ stand for slice-selection, readout, and time-encoding gradients, respectively. The crusher gradients remove artefacts from the images, the shift gradient reverses the detection order, leading to identical echo times *TE* for all echoes. The spin-packet re-phasing gradient un-winds the phase accumulated during spin-packet excitation.

Another variant of the time-encoding sequences is introduced in Figure 6a. All spin-packet-selective gradient pulses in Figure 6 are positive, in contrast to Figure 2 where the effect of positive gradients is compensated by negative gradients. Therefore, a spin packet which is excited by the *n*^th^ excitation pulse in Figure 6a is de-phased in the time-encoding dimension by all subsequent gradient pulses starting with the (*n* + 1)^th^ gradient pulse. In other words, the constant time-encoding gradient that is active during the detection interval in Figure 6a has the same function as the negative time-encoding gradient pulses in Figure 2 that are applied during excitation, since they re-phase the magnetization in the time-encoding direction.

In analogy to RASER experiments with constant spatial encoding gradients^20^, the image reconstruction must be adapted to the present case: before performing a Fourier transformation of each echo, a time-dependent linear phase correction φ has to applied to the data points according to the following equation^20^.

2 $$\phi =-\,2\pi {n}_{ro}\frac{\gamma {G}_{te}{{\rm{FOV}}}_{{\rm{te}}}}{\gamma {G}_{ro}{{\rm{FOV}}}_{{\rm{ro}}}}(\frac{{n}_{te}}{{N}_{te}}-\frac{{x}_{centre}}{{{\rm{FOV}}}_{{\rm{te}}}}-0.5)=-\,2\pi {n}_{ro}\frac{b{w}_{te}}{b{w}_{ro}}(\frac{{n}_{te}}{{N}_{te}}-\frac{{x}_{centre}}{{{\rm{FOV}}}_{{\rm{te}}}}-0.5)$$

where *G*_*te*_ and *G*_*ro*_ are time-encoding and readout gradients, FOV_te_ and FOV_ro_ are the fields of view in the time-encoding and readout directions, and *bw*_*te*_ and *bw*_*ro*_ are the bandwidths in the time-encoding and readout directions. The index *n*_*ro*_ refers to the *n*^th^ data point in the readout direction, while the index *n*_*te*_ refers to *n*^th^ data point in the time-encoding direction. The total number of time-encoding rows is *N*_*te*_ (equal to the number of excited spin packets), and x_centre_ is the position (with respect to the centre of the gradient coil) of the spin packet excited for *n*_*te*_ = *N*_*te*_/2. Such a phase correction is required because the time-encoding gradient that is active during data acquisition leads to a phase shift of the frequency-encoded signals.

**Figure 7:**
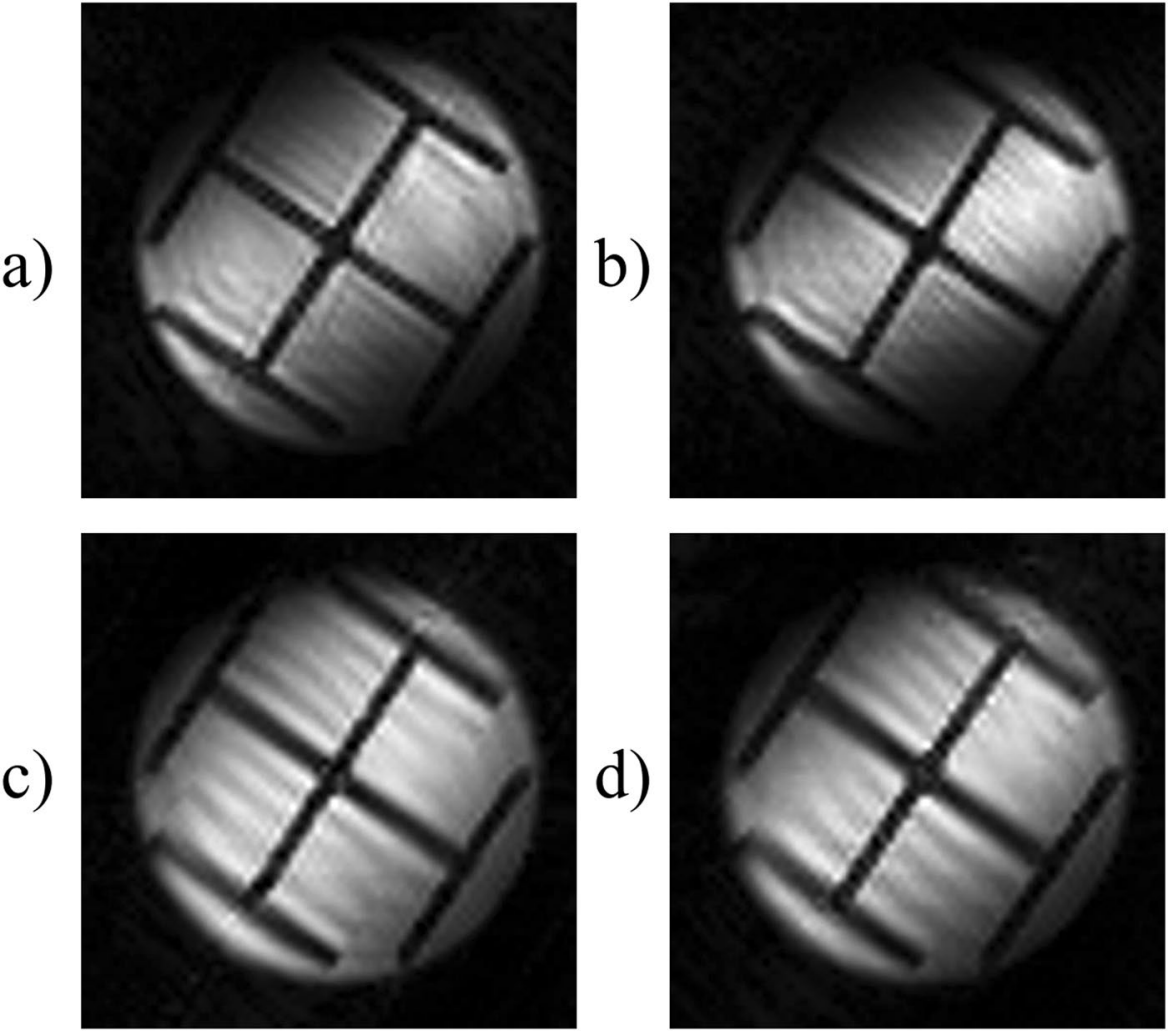
Four different images obtained using the sequence shown in Figure 6a for (**a**) and (**c**), and using the sequences of Figure 6b for (**b**) and (**d**). All images were obtained with a total matrix size of 64 × 64 with four-fold interleaving, an FOV of 27.0 × 27.0 mm, and *TE* = 14.4 ms. The lengths of the excitation pulses were 384 µs in all cases. In (**a**) and (**b**) the amplitude of the constant time-encoding gradient was 13.6 Gcm^−1^ = 0.136 Tm^−1^ causing significant signal losses due to diffusion, while in (**c**) and (**d**) the amplitude of this gradient was attenuated to 6.8 Gcm^−1^ = 0.068 Tm^−1^ to decrease signal losses.

Further reduction of the gradient switching rate can be achieved by replacing the excitation gradient pulses in the time-encoding direction in Figure 6a by a constant gradient that is active during the entire excitation period. The resulting sequence is depicted in Figure 6b. This will increase the total area of the time-encoding gradient in the excitation block. Consequently, the area of the time-encoding gradient in the detection block must be adjusted accordingly. Images obtained with the sequences shown in Figure 6 are presented in Figure 7. In Figures 7a and b, a large time-encoding gradient was used. The signal attenuation near the top-left and bottom-right corners of the images is due to diffusion. Note that these artefacts appear along an oblique axis, since two orthogonal gradients are applied simultaneously. These artefacts are absent in Figures 7c and d where we have used weaker time-encoding gradients.

### Removing non-uniform *T*_2_^*inh*^ effects

**Figure 8:**
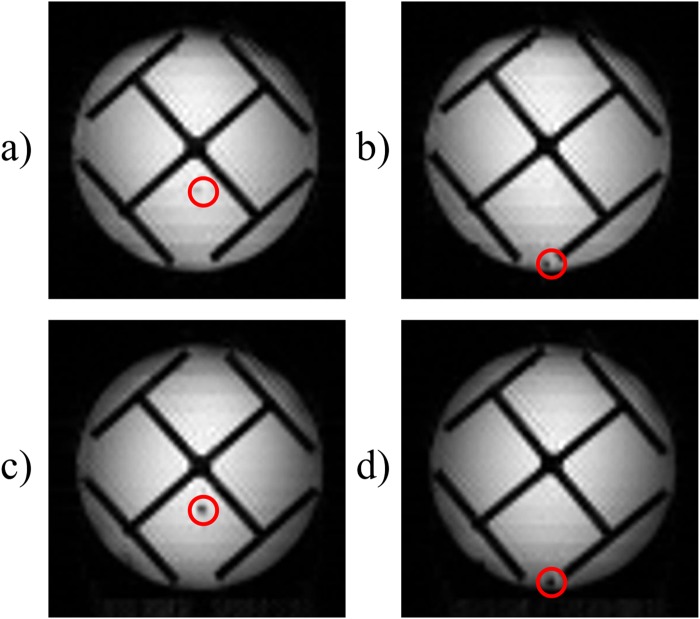
Four different images of a phantom of a cross-shaped piece of plastic immersed in water with a thin glass fibre, the tip of which is positioned within the thickness of the selected slice at the coordinates marked by red circles. Images (**a**) and (**b**) were obtained using the sequence shown in Figure 2b, and those in (**c**) and (**d**) using the sequences of Figure 9. The glass fibre can be seen in (**b**), (**c**) and (**d**), however, it is barely visible in (**a**) since *T*_*2*_^*inh*^ effects are refocused. Note that in (**c**) and (**d**) *T*_*2*_^*inh*^ effects are partially refocused to the same extent in all echoes across the entire object. All images were obtained with a total matrix size 64 × 64 with four-fold interleaving, an FOV of 27.0 × 27.0 mm, and *TE* = 14.4 ms. The lengths of the excitation pulses were 256 µs in all cases.

One drawback of the sequence of Figure 2b is that different parts of the object are affected by the inhomogeneous part of *T*_2_^*^ (which is defined by 1/*T*_2_^*^ = 1/*T*_2_ + 1/*T*_2_^*inh*^) in a non-uniform manner. For spin packets located near the edges of the field of view, the de- and re-phasing intervals before and after the π pulse have different durations. Therefore, the signals of these areas are significantly affected by *T*_2_^*inh*^ effects. On the other hand, for spin packets located near the centre of the field of view, the π pulse will refocus *T*_2_^*inh*^ effects. The effects of this non-uniform signal weighting can be appreciated in Figures 8a and b that were obtained using the sequence of Figure 2b. The phantom used for these experiments was the same as the one described in Figure 3, but with the insertion of a thin glass fibre made by stretching a piece of molten glass heated in a flame. The glass fibre was positioned so that its tip lies within the slice that is to be imaged. Since the glass fibre is very thin, the spin density is not significantly affected (less than about 3%) in the vicinity of the glass fibre, and the only effect on the image is due to susceptibility variations^22^. In Figure 8a, the glass fibre was located close to the centre of the phantom, where *T*_2_^*inh*^ effects are refocused, so that one barely can detect any evidence of the presence of the glass fibre in the image (its location is indicated by a circle). In Figure 8b, on the other hand, the glass fibre was located near the edge of the field of view, where *T*_2_^*inh*^ effects are not fully refocused, so that susceptibility effects lead to a dark spot.

**Figure 9:**
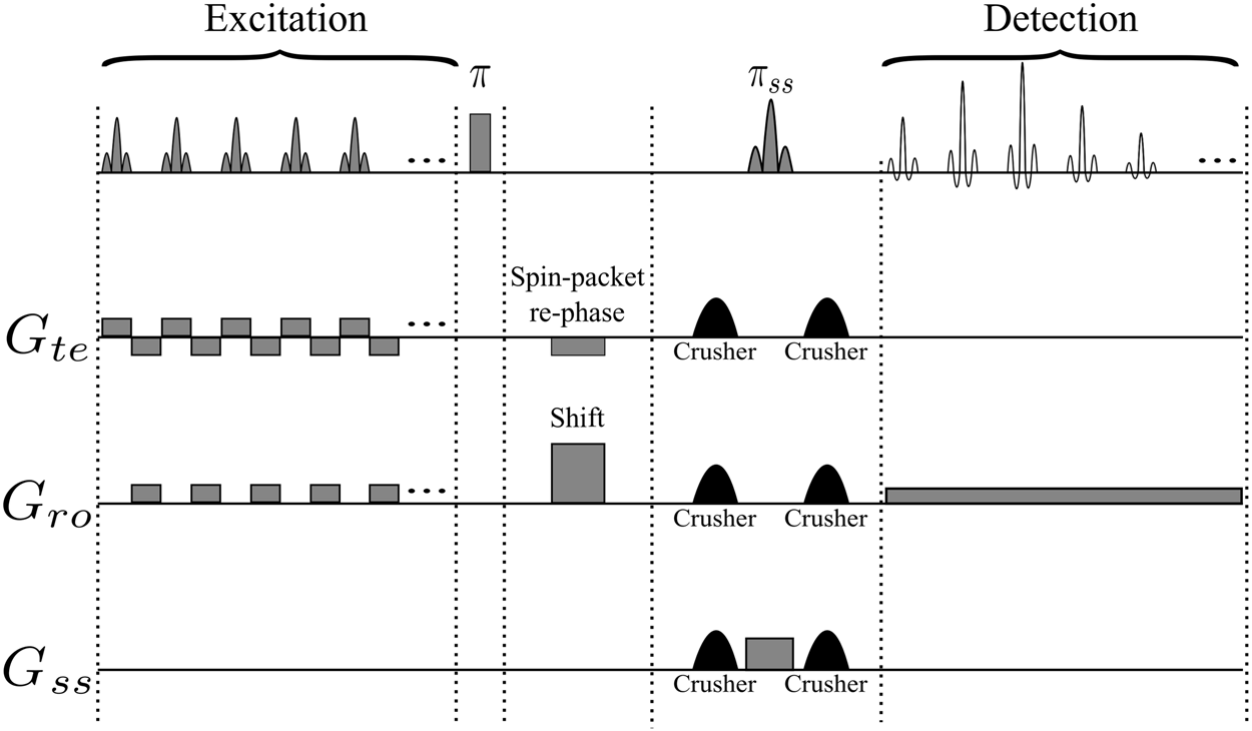
Modified time-encoding pulse sequence that leads to a uniform *T*_2_^*^-weighting across the entire object. The rectangular “hard” π pulse ensures that the effects of field inhomogeneities are the same for all spin packets. Since this π pulse is not applied immediately before the slice-selection pulse π_ss_, *T*_*2*_^*inh*^ effects will be partially refocused. The shift gradient pulse reverses the order in which the echoes appear, so that *TE* is identical for all echoes. Spin-packet re-phasing unwinds the phases accumulated during the excitation of the spin packets. *G*_*ss*_, *G*_*ro*_, and *G*_*te*_ stand for slice-selection, readout, and time-encoding gradients. The crusher gradients remove artefacts from the image.

To overcome this problem, one may use the sequence Figure 9 which comprises an additional rectangular non-selective π pulse. Consequently, all echoes in Figure 9 are affected uniformly by *T*_2_^*inh*^. If this π pulse were immediately adjacent to the selective π_ss_ pulse used for slice selection, there would be no net refocusing. However, it can be applied before the slice selection pulse π_ss_ so that it partially refocuses *T*_2_^*inh*^ effects^22^. The results of this modification are shown in Figures 8c and d, where the glass fibre appears in both images, regardless of whether it is located close to the centre or to the edge.

In the images of Figures 5, 7, and 8, weak stripes can be observed, which are due to the interleaving. Specifically, these stripes are due to attenuation factors (such as losses caused by diffusion) that depend on the timing of different echoes. The echo times of *n* consecutive rows (where *n* is the number of interleaved experiments, *n* = 4 in our case) are equal. Hence, *n* consecutive rows are affected in an identical manner by losses, while there may be a discontinuity between adjacent sets of *n* rows. In the experiments that are not interleaved this change in intensity occurs gradually.

It is also worth mentioning that these *T*_2_^*inh*^ effects contribute mainly to the contrast, and not to image distortions, which are mainly determined by the ratio of the internal field gradients to the external gradients used for the imaging sequence.

Although careful adjustment of the timing of MRI sequences is important for almost all experiments, the timing is particularly crucial for time-encoding with reduced switching rates^23^ (Figure 6). Indeed, for such time-encoding sequences (Figure 6), complete echoes only occur if the magnetization vectors are properly re-phased in *both* time-encoding and readout directions. Otherwise, the echoes are attenuated because the magnetization is still de-phased in one direction, thus reducing the SNR, and some artefacts may appear in the images. Since different gradient coils have their own characteristic behaviour, and since each combination of *rf* excitation pulses and gradient pulses that are used for spin packet excitation causes a unique de-phasing in the time-encoding direction, careful adjustment of the de- and re-phasing intervals in both directions requires that one first estimates the required amplitudes of the gradients. These amplitudes must then be slightly adapted empirically to achieve an image with the best quality. However, the settings do not need to be changed from one object to another when the experimental parameters are the same.

## Methods

All of our experiments were performed on a Bruker 800 MHz NMR instrument equipped with accessories for micro-imaging. The phantom was a cross-shaped piece of plastic that was placed in a 25 mm O.D. glass tube filled with de-ionized water. This phantom was used with a 25 mm imaging probe. The pulse programmes were written and implemented using the Topspin programme, and the data were processed by a home-written Python code. In all cases, the slice thickness was about 1 mm, the FOV was 27.0 × 27.0 mm, and the total matrix size was either 64 × 64 in both dimensions, or 64 in the *k*-encoding dimension, and 32 in the time-encoding dimension. In experiments with four-fold interleaving, the total FOV was 27.0 × 27.0 mm, and the total matrix size was 64 × 64; thus, the matrix size for each of the four complementary scans was 64 in the *k*-encoding direction, and 16 in the time-encoding direction. Other parameters are given in the figure captions. In time-encoding experiments designed to obtain uniform *T*_2_^*inh*^ effects, a thin glass fibre was positioned in such a way that the tip of the glass fibre ended within the thickness of the imaged slice. The glass fibre was made by stretching a piece of glass molten in a flame.

## Conclusions

It has been shown that the original version of the time-encoding sequence can be improved in several aspects. By inserting an additional gradient pulse, we were able to reverse the order in which the echoes appear. This leads to identical echo times for all echoes, and hence to a uniform signal attenuation due to relaxation, and also to a reduction on average of the signal attenuation due to diffusion. Furthermore, it has been shown how one can implement time-encoding sequences in an interleaved fashion in order to reduce the number and area of the gradient pulses and hence reduce signal losses due to diffusion. Moreover, it has been shown how one can reduce the switching rate of the gradients by rearranging positive and negative gradients. Finally, we have shown how one may obtain a time-encoding sequence where all echoes are uniformly affected by *T*_2_^*inh*^ effects.
